# Challenges and strategies to implement exclusive breastfeeding in the selected Districts of Limpopo Province, South Africa: professional nurses´ perspectives

**DOI:** 10.11604/pamj.2023.46.75.31408

**Published:** 2023-11-02

**Authors:** Azwinndini Gladys Mudau, Jabu Tsakani Mabunda, Lindelani Fhumudzani Mushaphi

**Affiliations:** 1 Department of Public Health, Faculty of Health Sciences, University of Venda, Thohoyandou, South Africa,; 2 Department of Nutrition, Faculty of Health Sciences, University of Venda, Thohoyandou, South Africa

**Keywords:** Exclusive breastfeeding, experiences, implementation, professional nurses

## Abstract

**Introduction:**

despite the efforts of professional nurses, exclusive breastfeeding rate is very low in South Africa. Per statistic South Africa, EBF rate is 32% with Limpopo Province being rated 8% as one of the lowest in South Africa. Implementation of exclusive breastfeeding required professional nurses´ efforts, lactating mothers, families, and community need information and support from the health care system. The purpose of this study aimed at exploring professional nurses´ challenges regarding implementation of exclusive breastfeeding in Limpopo Province.

**Methods:**

qualitative research involving phenomenological design was employed to explore professional nurses´ challenges using one on one, unstructured, in-depth interview with 30 professional nurses; 27 females and three males, working in the six birthing facilities of two selected districts; Vhembe and Waterberg, in Limpopo Province.

**Results:**

the three higher order themes emerged were challenges experienced by nurses during promotion of exclusive breastfeeding; findings revealed challenges as cultural and religious beliefs, lack of family/community and health professional support, teen and HIV positive mothers, nurses found to have challenges to implement exclusive breastfeeding. Healthcare system and services such as shortage of staff and lack of proper training on lactation management and perceived measures to promote exclusive breastfeeding suggested by health professionals, which include ongoing health education, door to door campaigns, implementation of policy by health care professionals would promote exclusive breastfeeding strategies that are in place and encourage families and community to support lactating mothers to improve their confident on breastfeeding.

**Conclusion:**

professional nurses had challenges in promoting exclusive breastfeeding. An intervention is needed to promote exclusive breastfeeding.

## Introduction

World Health Organization (WHO) and the United Nations Children's Funds (UNICEF) recommend that breastfeeding must be started immediately after birth, within 30 minutes, and to continue with breastmilk only for the first 6 months of infant´s life, before the introduction of solid food and continued breastfeeding until two years [1]. Implementation of exclusive breastfeeding (EBF) involve professional nurses, lactating mothers, families, and community need information and support from the health care system. Efforts were made through different strategies such as integrated management of childhood illness (IMCI), mom connect and lactating mothers support groups to promote EBF [2]. However, South Africa continues to experience high mortality among children under five years of age due to poor feeding practices which lead to paediatric dangerous diseases such as diarrhoea, upper respiratory infection, and malnutrition. It is known that up to 90% of under-five deaths could be prevented through implementation of EBF [3], however poor implementation of EBF has hinders its effectiveness at reducing child mortality. In South Africa, EBF rate currently stands at 32% with Limpopo Province being rated at 8% one of the lowest in South Africa which falls short of the 90% rate estimated as needed for maximal impact on reducing child mortality [4]. In South Africa, professional nurses give health education about EBF during antenatal and initiate EBF within 30 minutes after birth and continue supporting lactating mothers during post-natal care [5], despite all these efforts the rates are still very low compared to the recommended 90% target by WHO. Per statistics South Africa, initiation was rated at 78-98%, while EBF being rated the lowest [4]. Many babies are given solid feeds between two and three months of age and even within few days of birth [5]. These poor feeding practices pose risk to poor health outcomes in both infant and young child, as well as during teen years [6]. A study conducted in Kwazulu-Natal in South Africa to identify barriers to EBF, indicated the influence of the community, elders, and grandmothers on EBF practice [7]. Another study conducted showed that most women perceived breastmilk insufficiency and that their infants were not being adequately fed with breastmilk only [8].

Findings from other studies revealed challenges such as negative perceptions of breastfeeding among younger women and girls, lack of knowledge, desire for social acceptance and pressure to maintain original body shape as factors influencing negative attitudes and resistance to EBF [9]. In the North-West Province, South Africa, young girls choose not to breastfeed in fear of damaging their bodies and breasts [10]. Traditional and cultural beliefs are also regarded as strong challenges in the implementation of EBF in South Africa. In the Northern Cape, traditional medicines such as ”Muthi we Nyoni´´ are given to the babies immediately after birth to protect them against witchcraft [7] and this study revealed that EBF implementation need support not only from health care system but also from families and community at large. Lactating mothers sometimes do not want to be advised by the health care professionals on what to do regarding their babies´ feeding, they usually want to take decisions by themselves or families on their infant's feeding [5]. Save the children has trained over 100 nurses of all categories and 200 home-based carers on the topic of breastfeeding in Limpopo Province, thereby increasing their capacity to provide quality infant healthcare and prevention of mother to child transmission of HIV (PMTCT) to increase the rate of EBF [10], midwifery training in New Zealand is a good practical example in relation to support for breastfeeding and Per the New Zealand College of Midwives, the important standard of practice for professional nurses is working with lactating mothers, sharing decision-making, understanding and responsibilities. New Zealand midwives take into consideration women´s cultural beliefs and provide practical advice and relevant information to lactating mothers and their families [11]. Previous research also shows that lack of knowledge, staff poor attitudes and mothers´ perceptions regarding EBF; culture, beliefs, and absence of resources, skills as well as lack of support from the family and high prevalence HIV contribute to poor performance in the implementation of EBF [12]. Despite the availability of cost-effective prevention measures, acute respiratory infections and diarrhoeal diseases remain among the leading causes of mortality, in 2015 an estimated 9% of global under five deaths were due to acute respiratory infection [2]. Malnutrition contributes to nearly half of death in children of infants worldwide due to poor feeding practices [6]. Current evidence indicates that 64% of the infants are dying due to malnutrition in South Africa with 30, 2% in Limpopo Province [6]. Therefore, this study aimed at exploring professional nurses´ challenges regarding implementation of EBF in Limpopo Province. Research question was “what the challenges and strategies to implement EBF in the selected districts of Limpopo Province”.

## Methods

**Study design:** a qualitative, phenomenological research design was used, which was explorative. The design was chosen as elicitation research for gaining new insights, discovering new ideas and increasing knowledge of phenomenon [13], challenges of professional nurses on the implementation of EBF.

**Study setting and population:** this study was conducted in Limpopo Province. The province has five districts namely: Vhembe, Mopani, Sekhukhune, Waterberg, and Capricorn. It has 478 health facilities. This number is made up of the following: 411 PHC clinics 28 health care centers, 30 district hospitals, five regional hospitals, two special and two tertiary hospitals. This study was conducted in two (Vhembe and Waterberg) of the five districts of Limpopo Province and focused on six health care facilities. Two districts were purposively selected for this study namely, Vhembe and Waterberg. The selection was based on the initiation rates disparities, Vhembe had the highest initiation rate of 99%, while Waterberg had the lowest with 78%. Health care facilities from the three selected districts were selected using simple random sampling. The fishbowl technique was used and this involved name of each health facility category (hospital, health centre or clinic) from each selected district. In this study, the target population were professional nurses working in the birthing units of the Limpopo Province whilst the accessible population were the professional nurses from the two selected districts. Convenience sampling technique was used to select eligible participants. Those professional nurses who met the criteria and were on duty during data collection were included in the study. The sample size of this study was 30 professional nurses.

**Data collection tool:** data were collected using an individual, unstructured, in-depth interview consisted of the central question: “*may you kindly share with me how you implement EBF in your facility”*. The researcher immersed herself in the space of the participants to gain insight into the context of the study. This helped the researcher to understand main issues that may affect the quality of data and to develop trust with participants. The researcher intensively involved and became a research instrument [14].

**Data collection procedure:** the researchers arranged with the health care facilities managers and invited professional nurses. An appointment was made to visit the participants at the time that they were free and able to participate. The study objectives, aims, and benefits were explained to the participants, they were told that participation is voluntary and only participants who were willing to participate were given consent forms to sign so that they form part of the study. The researcher conducted one on one interview in a private place at the participant's convenient time. The duration of the interview was 30 to 45 minutes per participant, allowing the researcher to access the deeper meaning of professional nurses´ responses. The researchers also used field notes that contained information such as non-verbal cues and other gestures observed. Interview were conducted in English. The audiotape was used to record the interview which transcribed verbatim for analysis purposes. A central open-ended question was posed to the participants, followed by probing.

**Data analysis:** testch´s steps of data analysis were used to profile the qualitative factors that emerged from the participants´ responses. This process entails coding responses to establish cluster quotations and family themes to concert primary data into information readable by users. The researchers interpreted and gave meaning of the data to be able to gain understanding of the study. The independent coder analysed transcripts, reviewed raw data, and recorded information as well as written field notes.

**Measures of trustworthiness:** the clear delimitation and explicit description of the methods that were employed was expected to aid for transferability especially to similar populations [13]. The following enhanced conformability: carefully planning the research process, design, sampling, and data collection; recording of the participants during interview; transcribing the raw data from the tape and analyze raw data and findings through contextualisation.

**Ethical consideration:** ethical clearance was sought from the University of Venda Ethics Committee. Permission to conduct the study was granted from the Department of Health, Limpopo Province, Vhembe, and Waterberg districts and from the managers of the health care facilities where the study conducted. The names of participants have no bearing on the findings of the study. Therefore, they kept confidential. The researcher made sure that the data from the respondents could not be linked to them, no names were used, and instead codes were used to identify the respondents, for example participant number one. The researcher made sure that the supervisors from the university and the researcher are the only ones with access to the respondents´ information. Participation depended on the free will of the participants, and they were informed of their right to withdraw from the study at any time, if they wish to. Research conducted in such a manner that participants were not harmed.

## Results

**Demographics:** a total of 30 professional nurses participated: 23 females and 7 males took part in the study. Age of participants were between 20-59 years; the dominating part was 40-49. Eight (8) participants were single followed by 13 married, 7 divorced and 2 widow. Participants working experience was between 5 and 25 years with 37 of the longest experience (Table 1).

**Table 1:** socio-demographic characteristics of participants

	Category	Frequency	%
**Age**	20-29	6	20
	30-39	4	13
	40-49	11	37
	50-59	9	30
**Marital status**	Divorced	7	23
	Single	13	43
	Married	8	27
	Widow	2	7
**Qualifications**	Diploma in general nursing Science (psychiatric, community, midwife) (college)	10	33
	Diploma in nursing science (University)	17	57
	Degree in nursing science (University)	3	10
**Working experience**	5 to 10	3	10
	11 to 15	6	20
	16 to 20	11	37
	21 to 25	10	33

**Themes and sub-themes:** the findings were laid together using three themes as main headings with sub-themes under each. The first theme was the challenges experienced by the Professional nurses in the implementation of EBF. The second theme was healthcare system and services. The third theme is Perceive measures to promote EBF suggested by health care professionals (Table 2).

**Table 2:** challenges experience by professional nurses in the implementation of exclusive breastfeeding

Themes	Sub-themes
Challenges experienced by health care workers on the implementation of exclusive breastfeeding	Lack of adherence and negative attitude towards exclusive breastfeeding by teenage mothers experienced based on various reasons; going back to school, breastfeeding in public; fear of becoming out of shape
	Socio and cultural influence interrupts attempt to maintain exclusive breastfeeding
	Lack of compliance to exclusive breastfeeding by HIV+ mothers; lack of knowledge on the benefits, management, and support from family members of HIV^+^ lactating mothers’ influences cessation of exclusive breastfeeding; late antenatal care bookings poor understanding by young mothers regarding exclusive breastfeeding
Healthcare system and services	Shortage of staff and lack of time during care provision blamed for the implantation of exclusive breastfeeding by nurses
	The lack of formal and continuous training for nurses is viewed as problematic for the promotion of exclusive breastfeeding
Perceive measures to promote exclusive breastfeeding suggested by health care professionals	Outreach programmes suggest improving exclusive breastfeeding and health related issues
	Improvement of the on-going health education programme to encourage exclusive breastfeeding and aspects suggested
	Establishment of support groups which will promote learning from one another exclusive breastfeeding suggested

**Theme 1; challenges experienced by nurses during promotion of EBF:** this theme comprised of five sub-themes namely: Lack of adherence and negative attitude towards EBF by teenage mothers experienced based on various reasons; going back to school, breastfeeding in public; fear of becoming out of shape; socio and cultural influence interrupts attempts to maintain EBF.

**Lack of adherence and negative attitude towards EBF by teenage mothers experienced based on various reasons; going back to school, breastfeeding in public; fear of becoming out of shape:** most of participants indicated that teen mothers are not adhering to EBF because of various reason which include going back to school, breastfeeding in public as embarrassment, maintaining body shape and lack of knowledge regarding benefits of EBF. Teen mothers mostly don´t have love for their infants because their pregnancies were unplanned, they don´t have even enough time with their infants due to their busy schedule; some of them, they feel ashamed of themselves of having a baby at young age, they don´t want to breastfeed in front of their friends. They also believe that breastmilk is not enough.

**Participant (P1) says:** *“…you know what, as nurses we are trying our level best to implement EBF, we teach every day here in our institution but we have got challenges; such as teenage mothers, they´ve got so many problems: they don´t want to breastfeed in public, they interrupt EBF because they want to go back to school and they want to maintain their body shape”*.

**Participant (P4) adds on the wrong attitude of young mothers:** *“Implementation of EBF is a challenge here, young mothers don´t want to breastfeed for cosmetic reasons- fear of becoming out of shape and belief of insufficient breast milk, you know what? Wearing tight clothes, the way we dress nowadays, can´t allow you to take out the breasts so they prefer to use formulas”*.

**This also supported by participant (P15) who says:** *“…young mothers particularly teenager mothers don´t have knowledge on the benefits of breastfeeding because they don´t have time to attend Antenatal care (ANC), they are always busy, is a challenge. “Teenage mothers don´t have authority to can make their decisions. They have to listen to their mothers or in-laws and follow their instructions of feeding practices”*.

**Participants (P10) express her experience by saying:** *“Teen mothers, most of them don´t their babies, lack of love to the babies and lack of knowledge of the benefits of breastfeeding”*.

**Another participant (P14) adds:** *“Another burning issue is teenage mothers, having a baby at young age, and stress from being seen by friend while breastfeeding is another factor that influence EBF among young mothers because if the mother is stressed, she can´t breastfeed”*.

**Socio and cultural influence interrupts attempt to maintain exclusive breastfeeding:** many participants alluded family, parent/parent-in-law and religion as the negative factors to EBF, myth about breastmilk. Parents and parents-in-law are the main influencer; they give instructions on baby feeding practices. Lactating mothers respect and follow their parents or in-law´s instructions. Myths about breastmilk, that expressed breastmilk is contaminated also influence implementation of EBF and that giving a baby expressed breastmilk is taboo. Infants should be given soft porridge mixed with herbs or anointing water for protection.

**Participant (P1) says:** *“Exclusive breastfeeding not done because of the influence from the in-laws. In laws or parents have an influence to encourage lactating mothers to go to traditional healers, the traditional healers are the ones who are forcing lactating mothers to give their infants other liquids and soft porridge mixed with herbs. “There is a myth because they think that expressed breast milk is contaminated, in our culture, everything coming from the human body is contaminated and giving it to the baby is taboo. Because our grannies do not have knowledge, they think expressed breastmilk is dirty and it can cause diseases like diarrhoea, some are Christian… they give their infants anointing water”*.

**Similarly, participant (P3), shares her experience by saying:** *“This is from my experience, the influence of family members is real, and it hinders implementation of EBF. Before I became a nurse, I couldn´t practice EBF because I was married, and my in-laws were the solution of the family, … I started mixed feeding three days after I was discharged from the hospital´´. “I mean, EBF was not practised because I was a new daughter in-law in the family, and I have to follow my in-law´s religion and culture of giving a baby anointing water at three days, culture and religion is very important to us black people’’. ‘‘Culture and beliefs, you know as black African people, we must do rituals for our babies, and we must give herbs mixed with soft porridge every morning to our babies as black people that is our culture. Religion is another factor; we must give anointed water every morning as instructions from pastors, even myself as a nurse, no ways culture is culture”*.

**Participant (P12) with the same views says:** *“culture is still a challenge, people are totally different, some are Bangladesh they prefer fresh milk, I think culture, religion and beliefs are the main factors including social aspects”*.

**Per participant (P18):** *“Exclusive breastfeeding not practiced because of the grandparents. When I go back to work, they started to mix feed believing that the baby is hungry, this is a challenge”*.

**Lack of compliance to EBF by HIV^+^mothers; lack of knowledge on the benefits, management, and support from family members of HIV lactating mothers´ influences cessation of EBF; late ANC bookings; poor understanding by young mothers regarding EBF:** participants alluded to challenges related to mothers´ illness that they thought influenced the implementation of EBF. HIV^+^ mothers be afraid of infecting their infants. Lack of knowledge and support from family members

**Participant (P3) says:** *“HIV status of the lactating mother can influence cessation of breastfeeding just like lack of knowledge on the benefits and management of EBF by lactating mothers and their families, Lack of support from your family, you know”*.

**Participant (P2) also add that:** *“Another challenge is those lactating mothers who are HIV? fear of transmission from mother to child”*.

**Another participant (P4) also says:** *“HIV^+^ mothers have problems, if viral load is high, they fear of infecting their babies; illness or other chronic conditions of the mothers like breast problems*.

**Participant (P5) with the same views alluded that:** *“…Mothers´ illness is a barrier, HIV^+^mothers, you can teach and give them information, but their status is the barrier”. “HIV mothers and chronic conditions mothers or those with breasts problems, HIV^+^ mothers still have that perception to say if they can breastfeed their infant, they will infect them with HI virus. I think lack of knowledge Participant (P5) continued*.

**Participant (P17) alludes to this notion by saying:** *´Mother and baby´s illness, especially HIV^+^mothers, I told you about late booking cases and support from family members,”*

**Theme 2; healthcare system and services:** this theme comprised of two sub-themes namely: shortage of staff and lack of time during care provision blamed for the implantation of EBF by nurses and the lack of formal and continuous training for nurses is viewed as problematic for the promotion of EBF.

**Shortage of staff and lack of time during care provision blamed for monitoring adherence of EBF by nurses:** institutional challenges found to be influencing the implementation of EBF were shortage of staff and time to teach, due to lack of support, knowledge by staff and attitude of staff. Participants alluded that they are short-staffed, they don´t have enough time to teach lactating mothers about EBF

**Participant (P4) says:** *“Shortage of staff, we don´t have time to give health education to our clients. We are short staffed and overworked. We teach but is not enough, that´s our challenge, you can´t teach while the que is too long waiting for you, that supermarket approach is disturbing, …*.

**Another participant (P2) also emphasises that:** *“We do have challenges, time to teach, workload and overworked, in our institution is worse, is not easy because we don´t have enough time to teach, and we are short staffed”*.

**Lack of formal and continuous training for nurses viewed as problematic for promotion programme, nurses´ attitudes:** lack of training of EBF among the staff was found to be another institutional challenge to the successful implementation of EBF. New professional nurses without training, they don´t have knowledge and recent information on lactating management.

**Participant (P11) points out that:** *“Although we don´t have time to teach the lactating mothers, we also didn´t receive any training on breastfeeding, I need just in-service training, I think I will do better´´*.

**Participant (P19) elaborates:** *“You know, lack of knowledge of health care worker, especially new professional nurses. They usually give wrong methods of family planning, the method that reduces the secretion of breastmilk, training on breastfeeding management will be better for them, and even old ones, yes, we need continuous training´´*.

**Per one participant (P6) who observes that:** *“Lack of information of professional nurses is our main challenge here, I can say we are not implementing EBF; we don´t have information; we don´t have time to read the policies. This is very much serious, as nurses we need training, new information to implement EBF”*.

**Participant (P10) says:** *“I think our attitudes as nurses is not good when it comes to teaching mothers the importance of EBF, especially after having been working under pressure. Some of the nurses do not have time to deal with pregnant women or lactating mothers who ignore instructions. They think they do it deliberately particularly those nurses who have not experienced pregnancy. When mother tell them, problems related to breastfeeding, they simply ignore or just act to dismiss the mothers. Teaching breastfeeding requires us to have focus and positive mind”*.

**Participant (P19) affirms the views expressed above when she says:** *“To implement EBF is not easy; We have got challenges here, some of the staff members´ attitudes. They just have negative attitudes towards giving health education. They are reluctant to give health education”*.

**Theme 3; perceive measures to promote EBF suggested by health care professionals:** professional nurses suggested several possible activities which were categorised into: outreach programmes for breastfeeding and pregnancy women; health education to the families and communities, Establishment of support groups which will promote learning from one another suggested.

**Outreach programmes suggested to improve EBF and other health related issues:** participants suggested door to door campaign to teach families; fathers, grandmothers/fathers, about the benefits of EBF; some participants alluded that awareness programmes, ongoing health education during ANC and establishment of support groups as of significant to promote EBF. Periodic visits to monitor EBF to lactating mothers and establishment of lactating mothers support groups.

**Participant (P1) perceives that:** *“…I think both strategies are working, but door to door is more effective because when visiting their household monthly, we find the grannies and they are the ones who are responsible and influence the mothers, so we give them information on the importance of EBF”*.

**The participant (P1) went on:** *‘‘Maybe by doing outreach and door to door health education, I mean to involve everyone in the family like fathers, grannies, and the whole family. We need to come up with something that can improve existing strategies and that everyone will be motivated. What I am suggesting is on-going education; lactating mothers can be motivated by rewards, like to have an event to encourage lactating mothers to do EBF and we reward those who are doing well on EBF’’ ‘‘Awareness programmes through media and health education during ANC, capacity building, periodic check on lactating mothers, and establish support group, promote mother mentorship. Yes, me, myself, I feel like giving health education to the primary level can promote EBF and establishment of support groups to the primary level is the best way”*.

**Participant (P14) also suggests:** *“Let´s try to do door to door campaigns or to follow-ups to lactating mothers after discharge to monitor breastfeeding for six months at home”*.

**Participant (P7) also suggests that:** *“May be to conduct outreach programmes such as door to door campaigns, teach about the benefits of EBF. To encourage those mothers who successfully manage to exclusively breastfeed their infants for six months to counsel others. We can also use mentor mothers to counsel mothers as we don´t have much time to share information with the mothers”*.

**Participant (P4) adds:** *“Oh yes, I think to empower woman with knowledge on the importance of EBF, to have breastfeeding ambassadors to encourage other lactating mothers, to encourage establishment of support group and home visits”*.

**Improvement of the on-going health education programme to encourage EBF and all related aspects suggested:** participants suggested that health education is the key to success. Community empowerment, teaching pregnant women and their families about the importance of breastmilk.

**Participant (P11) add:** *Teaching families and communities to support lactating mothers, door to door campaign or to teach pregnant women along with their parents or relatives during ANC”*.

**The participant (P19) says:** *“Ok, lets continue to give health education to the community and family maybe that old strategy of visiting lactating mothers after discharge to monitor breastfeeding and hygiene; yes, I can recommend that strategy it was effective by then. Let´s visit lactating mothers during puerperium”*.

**Participants (P18) supported the above knowledge by saying:** *“I think health education is a way to go, we don´t teach them sufficient information, I think to invite family members with the pregnant women during ANC and teach them, lets involve community and families”. “Yes, it is our duty, we need to teach parents and in-laws about breastfeeding to support lactating mothers and maybe campaign will also help to put across the message to them” …*

**Participant (P3) says:** *“Health education to the lactating mothers and families is the key to success, teaching them about feeding practices” ..*.

**Establishment of support groups which will promote learning from one another regarding EBF suggested:** support group was suggested as one strategy that can influence EBF. Lactating mothers coming together and share their experiences and identification of EBF ambassadors.

**Participant (P1) says:** *“During ANC, we should let pregnant women and lactating mothers support each other. Pregnant women and lactating mothers should be allowed to come together and encourage each other on EBF and other issues to do with babies. I think pregnant women are eager to learn from breastfeeding mothers at the same time breastfeeding mother like to talk about their experiences”*.

**Participant (P10) believes that:** *“To establish support group, to identify EBF ambassadors to encourage others and to award those who manage to maintain EBF”*.

## Discussion

The WHO and UNICEF recommend: exclusive breastfeeding for the first six months of life, introduction of solid foods at six months together with continued breastfeeding up to two years of age or beyond and breastfeeding in the context of HIV [3]. However, many infants do not receive EBF. Only about 36% of infants were exclusively breastfed over the period of 2007-2014 [3]. This conforms to statistics of South Africa where only 32% initiative EBF with Limpopo Province rated the least at 8% in the country, contradicting WHO´s target of 90% [4]. In this study, professional nurses were given an opportunity to express their challenges on the implementation of EBF. The main challenges identified in the study indicated understaffing, low facilities and poor skills of professional nurses. Provision of adequate information to pregnant women and lactating mothers to promote EBF relies on adequate workforce. Limpopo Province is facing a serious resources' shortage in the health care facilities. Because of heavy workload due to shortage of staff, professional nurses reported poor performance in implementing EBF. Shortage of resources therefore poses a challenge to EBF by hindering the provision of support to enable mothers to breastfeed exclusively.

Cultural and religious beliefs, and parents or parent´s in-law are regarded as major challenges. Infants are given soft porridge mixed with herbs or anointing water for protection as early as from three days post-delivery. Young mothers gave birth before they are ready, going back to school, fear of losing their body shape, breastfeeding in public is considered as an embarrassment. Teen moms don´t have time to breastfeed and shows love to their infants [15]. HIV status was found to be responsible for a decrease in the duration and exclusivity of breastfeeding. Professional nurses alluded that mother´s illness is a challenge in the implementation of EBF. This is confirmed in the study conducted in Uganda which found a positive association between decreased duration of breastfeeding and positive status of the mother due to fear of mothers transmitting the virus to their infants [9]. Fear of transmission of HIV? mothers to their infants was alluded as a major hindrance to EBF. In the study done on perception of the role of maternal nutrition in HIV? lactating breastfeeding women, it was reported that most women perceived EBF as a factor that may increase the progression of HIV [12]. In terms of the women, studies have also supported that many HIV lactating women lack EBF knowledge and support [1,6]. Such practices predispose people to poor health outcomes in both their infant and young child years, as well as adulthood. Some do not know the benefits while others are ignorant for some personal reasons [6].

Professional nurses mentioned several measures of implementing EBF. Among a myriad, outreach program, health education at primary level and door to door campaign was mentioned. Although mother mentorship is ongoing in most regions of the area, but some professional nurses stressed a need for reinforcement. They also suggest active education and monitoring. There was a need to increase the capacity of nurses by train and motivate them to be committed in the drive for EBF. The measures of promoting EBF in the study conforms to several studies. For instance, the Baby Friendly Hospital Initiative (BFHI), a global initiative aimed at creating a health care environment that is promotive, protective, and supportive of breastfeeding, this initiative suggests the provision on information session for all pregnant women, assist lactating mothers to initiate breastfeeding, avoid supplements and the establishment of breastfeeding support groups. Unlike many other measures, this study identifies need for door to door campaign and capacity building, periodic check on lactating mothers and awareness programs [8]. South Africa also suggests that all professional nurses working with children under the age of five years need to train in IMCI, they need encouragement and support to use its tools as a resource for EBF practices amongst women and a more serious breastfeeding policy to support the drive [7].

## Conclusion

The research study was about challenges and strategies to implement Exclusive Breastfeeding in the Selected Districts of Limpopo Province, South Africa: professional nurses’ perspectives. Breastmilk only is enough to child growth. It is the best feeding practise for infants. This study found that the majority of lactating mothers stopped breastfeeding as early as before the infant could be six months old. The study also highlighted that there is inadequate support provided to lactating mothers by professional nurses and families. In addition, there is also a marked lack of literature regarding support of professional nurses in the implementation of EBF in the birthing facilities' context. Despite availability strategies and policy to promote EBF which supposed to be implemented by the professional nurses the rate is low.

### What is known about this topic

- *Professional nurses had challenges in promoting EBF among lactating mothers because of their cultural and religious beliefs, teen mothers, HIV status of the lactating mothers;*
- *A shortage of staff, coupled with a lack of professional development about EBF promotion and support*.

### What this study adds

- *Unlike many other measures, this study identifies need for door to door campaign and capacity building, periodic check on lactating mothers for six months post-delivery and awareness programmes;*
- *The study also highlights the need to strengthen family and community support to promote adoption of EBF*.
